# Optimization of sample preparation and green color imaging using the mNeonGreen fluorescent protein in bacterial cells for photoactivated localization microscopy

**DOI:** 10.1038/s41598-018-28472-0

**Published:** 2018-07-04

**Authors:** Iris Stockmar, Helge Feddersen, Kimberly Cramer, Stephan Gruber, Kirsten Jung, Marc Bramkamp, Jae Yen Shin

**Affiliations:** 1 Munich Center for Integrated Protein Science (CIPSM) at the Department of Biology I, Microbiology, Ludwig-Maximilians-Universität München, Martinsried, Germany; 2 Department of Biology I, Microbiology, Ludwig-Maximilians-Universität München, Martinsried, Germany; 3 Department of Fundamental Microbiology, University of Lausanne, Lausanne, Switzerland; 4 Present Address: Max Plank Institute for Biochemistry, Martinsried, Germany

## Abstract

mNeonGreen fluorescent protein is capable of photo-switching, hence in principle applicable for super-resolution imaging. However, difficult-to-control blinking kinetics that lead to simultaneous emission of multiple nearby mNeonGreen molecules impedes its use for PALM. Here, we determined the on- and off- switching rate and the influence of illumination power on the simultaneous emission. Increasing illumination power reduces the probability of simultaneous emission, but not enough to generate high quality PALM images. Therefore, we introduce a simple data post-processing step that uses temporal and spatial information of molecule localizations to further reduce artifacts arising from simultaneous emission of nearby emitters. We also systematically evaluated various sample preparation steps to establish an optimized protocol to preserve cellular morphology and fluorescence signal. In summary, we propose a workflow for super-resolution imaging with mNeonGreen based on optimization of sample preparation, data acquisition and simple post-acquisition data processing. Application of our protocol enabled us to resolve the expected double band of bacterial cell division protein DivIVA, and to visualize that the chromosome organization protein ParB organized into sub-clusters instead of the typically observed diffraction-limited foci. We expect that our workflow allows a broad use of mNeonGreen for super-resolution microscopy, which is so far difficult to achieve.

## Introduction

Optical super-resolution microscopy techniques such as photoactivatable localization microscopy (PALM)^1^ are commonly used to describe the *in situ* organization of macromolecules — *e*.*g*. proteins and nucleic acids— with spatial resolution approximately 10 times higher than diffraction-limited microscopy^2–4^. Although this impressive improvement in spatial resolution has already enabled many new biological discoveries^5^, implementing super-resolution imaging in practice is not trivial and requires significant expertise for trouble-shooting and experimental optimization. Here, we contribute practical advice to accomplish the challenging task of PALM imaging in the green channel.

One of the key features of PALM is that it employs genetically incorporated fluorescent proteins (FP) to label target proteins, which has the advantage of ensuring that each protein carries exactly one fluorophore^6^. Spectrally distinct photoactivatable fluorescent proteins (PA-FPs) make multi-color super-resolution imaging feasible, allowing scientists to structurally describe more than one cellular component at a time^7–10^. Dual-color PALM using an orange and a green PA-FPs is such an example, and it has been successfully employed to describe protein distribution in various eukaryotic cells^10–14^, and to lesser extend in bacterial cells^15–18^, most likely due to challenges in employing green PA-FPs. On the other hand, the functionality of target proteins often is compromised by genetically incorporated FPs, so it can be difficult to find a suitable tag. Therefore, more options of green PA-FPs for PALM can allow scientists to conduct studies that were previously not feasible.

mNeonGreen has been widely utilized to investigate biological processes in various organisms, including bacterial and eukaryotic cells, as well as mice^15,19–24^. Although the developers of mNeonGreen have already shown its use for super-resolution PALM microscopy, and its improved performance over other green fluorescent proteins for PALM (*e*.*g*. Clover, mEGFP and mEmerald)^25^, mNeonGreen has still been mainly utilized by diffraction-limited microscopies. Some of the key features that make mNeonGreen a promising option for PALM are its capability to photo-switch, high brightness, high photostability and short maturation time^25^. The latter being particularly crucial to study proteins in fast-dividing organisms such as bacteria and yeast.

**Figure 1:**
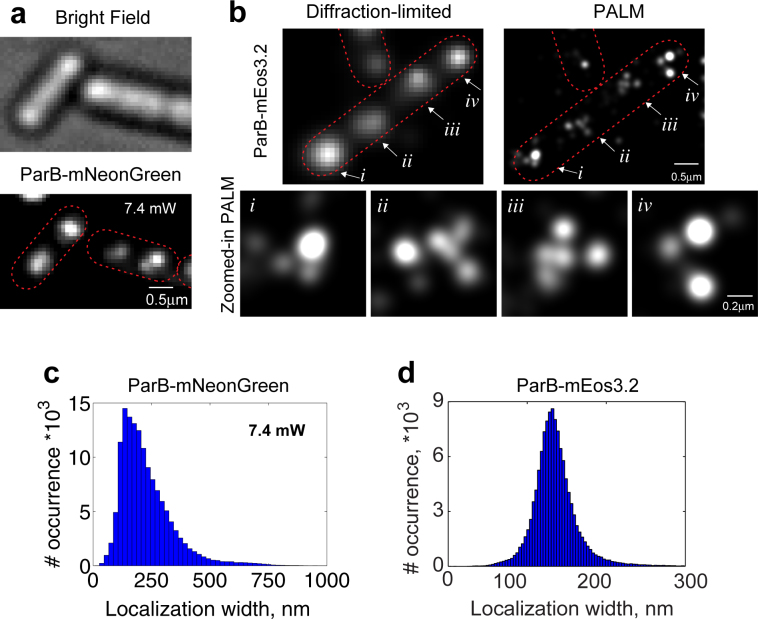
PALM images of *B*. *subtilis* ParB fused to mNeonGreen or to mEos3.2. *Bacillus subtilis* expressing **(a)** ParB-mNeonGreen (BSG2204) or **(b)** ParB-mEos3.2 (BSG2205) were prepared and imaged as described in Materials and Methods. **(a)** ParB-mNeonGreen expressing cells were imaged with pseudo-TIRF illumination with moderate 488 nm laser power. **(b)** ParB-mEos3.2 expressing cells were imaged with pseudo-TIRF illumination with 405 nm and 561 nm laser. A representative cell is shown as artificially constructed diffraction-limited image from the PALM data. Regions of interest (i to iv) from the PALM image are shown zoomed in below. Data were analyzed using the Zen software (Zeiss) (see Materials and Methods for details). Red dotted lines delineate cells. **(c)** Histogram of the ParB-mNeonGreen localization width at 7.4 mW (15%) of 488 nm power and **(d)** Histogram of the ParB-mEos3.2 localizations width. Localization width is the radius at which the fitted Gaussian drops to e^−1^ of its maximum. Brightness of the PALM image are adjusted to emphasize description of the structure.

Commonly used green PA-FPs (*e*.*g*. Dronpa and its mutants) typically require photo-manipulation with violet and blue lights, which drive the molecule back and forth between the bright and dark states, respectively^26^. The quality of PALM data acquisition critically depends on the tight modulation of these back and forth processes^27^. For instance, understanding the photophysical properties of Dronpa was critical to enable Dronpa-based PALM imaging^27^, as well as to develop improved green PA-FPs such as mGeos^14^ and rsKame^10^. mNeonGreen also switches back and forth between the two states, bright and dark^25^. However, contrary to Dronpa, the switching process is controlled only by one light wavelength (blue), which introduces artifacts in PALM images if post-data processing is not applied^15^ (Fig. 1). Unfortunately, the switching mechanism is not well understood, which can be one of the reasons that prevents scientists from using mNeonGreen for PALM imaging on a regular basis.

In this work, we sought to utilize mNeonGreen for PALM imaging to study the organization of bacterial proteins. Our first attempt for such imaging was unsuccessful due to the simultaneous emission of multiple nearby mNeonGreen molecules, resulting in overlap of their point spread function. Consequently, during image processing these overlapping emitters were fitted as single molecules, which generated localization centers far from the actual molecule positions. Therefore, to develop a method to truly enable mNeonGreen for PALM, we have characterized the properties of mNeonGreen, evaluated the effect of illumination power on image quality and developed a simple data filtering step using the temporal and spatial information of the detected localizations. In addition, to preserve the cellular structure under study as well as the fluorescence signal, we have optimized each of the sample preparation steps.

Finally, we applied our PALM imaging workflow for mNeonGreen to visualize the bacterial cell division protein DivIVA, and we observed the expected double band at the division sites, which can only be seen with super-resolution microscopy^28^. Furthermore, we also applied our method to visualize the bacterial chromosome organization protein ParB and instead of the typically described focus^29,30^, we observed sub-clusters. The biological relevance of the sub-clusters is under study.

## Results

### Imaging mNeonGreen with super-resolution microscopy PALM

The photo-switching capability, fast maturation time as well as high quantum yield and brightness are some of the features that make mNeonGreen attractive for PALM imaging^25^. Yet, our initial attempt to produce sub-diffraction-resolution image of the bacterial protein ParB with mNeonGreen was unsuccessful.

ParB is a bacterial protein that binds specifically to the centromeric *“parS”* sites distributed along the chromosome. Self-assembly and binding of ParB to specific and non-specific sites of the DNA presumably participate in DNA segregation^31,32^ as well as help folding the chromosome into multiple and small domains^33^. Because *Bacillus subtilis* cells contain 8 *parS* sites, it is expected that a ParB focus is composed by multiple sub-clusters, which has been visualized with structured illumination microscopy (SIM) super-resolution microscopy^34^. However, our PALM images of ParB-mNeonGreen did not show sub-clusters (Fig. 1a), and instead showed foci similar to the ones obtained with diffraction-limited microscopy^30,35^ (Fig. 1b, top left).

One could interpret these ParB foci as the true representation of ParB organization. On the other hand, the lack of sub-clusters could be an artifact of the imaging technique. To investigate these two possibilities, we constructed a new *Bacillus subtilis* strain that expresses ParB fused to mEos3.2. Both strains expressing either ParB-mNeonGreen or ParB-mEos3.2 showed a normal cell growth (Supplementary Fig. 5). Diffraction-limited microscopy showed that these cells contained ParB foci along the cells at the expected locations (Fig. 1 and Supplementary Fig. 6). These results together indicate that neither mNeonGreen nor mEos3.2 impaired the function of ParB.

Next, we imaged cells expressing ParB-mEos3.2 with PALM. Interestingly, ParB-mEos3.2 showed that the diffraction-limited focus is indeed composed by multiple sub-clusters (Fig. 1b), somewhat similar to the previously observed ParB images with SIM^34^, which is in agreement with what we have initially expected. Thus, our PALM results of ParB-mEos3.2 support the notion of chromosome folding into multiple domains and suggest that mNeonGreen could not provide high-quality super-resolution imaging with a naïve (or standard) approach.

### Effect of the illumination power on simultaneous activation of mNeonGreen

**Figure 2:**
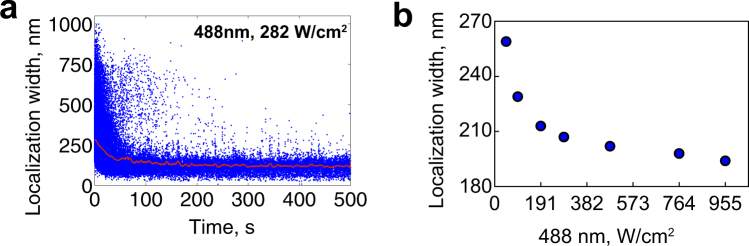
Effect of the laser power on the width of detected mNeonGreen localizations. (**a**) Individual mNeonGreen localization width over time from the Fig. 1a data. Red line is a moving window average of 100 frames. **(b)** Mean localization width of mNeonGreen at various 488 nm laser power (46, 91, 191, 282, 458 and 763 W/cm^2^, corresponding to 2.5, 5, 10, 15, 25 and 40% in the Zen software). Each data point represents the mean value from hundreds of localizations at the given laser power. Localization width is the radius at which the fitted Gaussian drops to e^−1^ of its maximum.

Our results show that the ability of resolving the sub-clusters of ParB domains depends on the utilized fluorescent protein (mNeonGreen or mEos3.2) when using a naïve imaging strategy (Fig. 1a,b). This discrepancy most likely arises from imaging artifact rather than from the actual ParB organization. Because PALM images are reconstructed from many single molecule localizations, we examined the properties of these localizations. Specifically, we inspected the localization width of the detected signal, which we report here as the radius around the localization center at which the fitted Gaussian function drops to e^−1^ of its maximum (note, that the reported width is $\sqrt{2}\sigma$, where $\sigma$ is the Gaussian standard deviation). As expected, the width of mEos3.2 localizations were normally distributed (Fig. 1d). Contrary to the expected normal distribution, the histogram of mNeonGreen localizations width showed a long tail at larger widths (Fig. 1c). These mNeonGreen localizations with larger widths most likely correspond to the overlapping images of multiple nearby mNeonGreen molecules emitting simultaneously. Consistently, those localizations with large widths appeared more pronounced at the beginning of image acquisition (Fig. 2a), indicating that localizations with large widths are most likely due to simultaneous photo-activation of multiple nearby molecules. Unfortunately, these simultaneously emitting molecules introduce artifacts in super-resolution images because the single fitted localization center is far from the multiple actual molecule positions.

Typically, photoswitching behavior of fluorescent molecules depends on the power of illumination. Therefore, we investigated the effect of various illumination powers on the localization width of detected mNeonGreen molecules. The width of localizations decreases (from ~260 nm to ~190 nm) with increasing laser power and reaches a plateau at moderate power (Fig. 2b and Supplementary Fig. 2 for full distribution). Thus, simultaneous photo-activation could be reduced with higher illumination powers, but not completely eliminated. Hence, we acquired our images with a moderately high power (15%).

### Comparison of photon budget and on- and off- switching rates of mNeonGreen and Dronpa

Next, we sought to investigate how mNeonGreen’s crucial properties for superresolution imaging, such as photon budget and on- and off- switching rates, compare to other green PA-FPs, specifically to mGeosM^14^ and to the commonly used Dronpa^36^. To evaluate these properties under conditions similar to superresolution imaging experiments, we constructed *Bacillus subtilis* strains, via allelic replacement, that express DivIVA fused to either mNeonGreen, Dronpa or to mGeosM.

**Figure 3:**
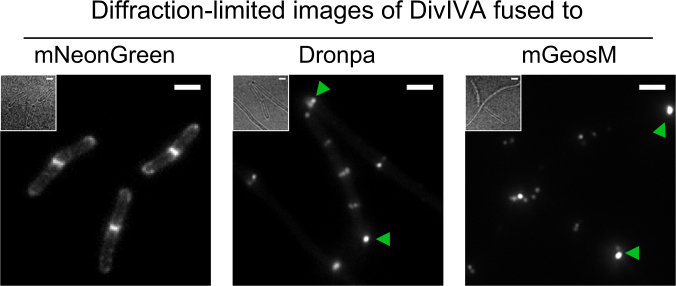
Diffraction-limited images of *B*. *subtilis* expressing DivIVA fused to either mNeonGreen, Dronpa or mGeosM photoactivatable fluorescent proteins. *B*. *subtilis* strains expressing DivIVA fused to mNeonGreen (BHF039), Dronpa (BHF057) or mGeosM (BHF058) from their native promoter were prepared and imaged as described in Material and Methods. Representative fluorescence and their corresponding bright field (inset) images of DivVIA fusion expressing cells are shown. Green arrows indicate mislocalized DivIVA fusion protein. Scale bars, 2 µm.

The diffraction-limited microscopy of these fusion protein expressing strains showed different degrees of DivIVA functionality, from mild to strong effect as follows: mNeonGreen, Dronpa and mGeosM (Fig. 3). Although less noticeable for the mNeonGreen fusion protein expressing strain, all strains show increments in cell length compared to their typical dimensions, similar to the *divIVA* deletion strain^37^. Both, Dronpa and mGeosM fusion protein distribution pattern shifted from the typical band at division septum to the cell poles as well as presenting multiple spots instead of bands (Fig. 3). The degree of mislocalization was higher for the mGeosM fusion protein strain. Contrarily, mNeonGreen fusion protein formed the expected band and localized at the expected division septum. Note that all three strains were constructed in the exact same way, but the functionality of DivIVA was affected in a different manner. Because mGeosM fusion protein was not fully functional, we thus did not further characterize this protein.

**Figure 4:**
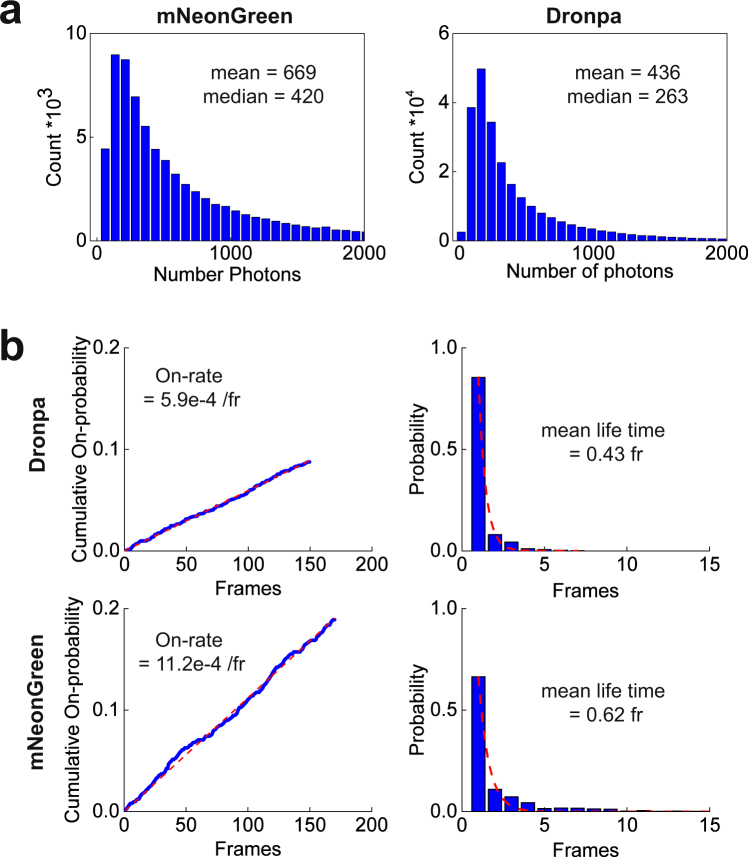
Photo-physical properties of mNeonGreen and Dronpa. Histogram of detected photon number for **(a)** mNeonGreen or Dronpa fused to DivIVA expressing *B*. *subtilis* strains (BHF039) or Dronpa (BHF057), respectively. Mean and median of the number of photons are indicated. **(b**, left) Initial linear section of the relative cumulative on-switching probability vs time was fitted (red dotted line) linearly to obtain the on-switching rate. **(b**, right) Distribution of the on-state lifetime was fitted to an exponential decay function (red dotted line) to obtain the mean life time, the inverse of which yields the off-switching rate. One frame corresponds to 20 ms. (See also Supplementary Table I).

The distribution of photon numbers detected per localization are shown in Fig. 4. Fewer photons were detected for Dronpa (mean: 436) compared to mNeonGreen (mean: 669), which is in good agreement with previous studies^25,38^. The on-switching rate for Dronpa was measured as described earlier^38^. Briefly, we started to acquire data on the DivIVA-Dronpa expressing strain in absence of the activation laser (405 nm) and in presence of imaging light (488 nm). Then, data acquisition was continued with simultaneous illumination of activation and imaging light until completion. The slope obtained from the total number of activation events accumulated in a period of time without activation light divided by the total number of activation events represents the on-switching rate. The off-switching rate was obtained from the inverse of the mean lifetime of the on-state (Fig. 4b and Supplementary Table I). The lifetime for Dronpa determined here (0.009 s) is in a good agreement with the previously determined value (0.0096 s)^38^. The mean lifetime for both, mNeonGreen and Dronpa, were similar (0.009 s and 0.012 s, respectively). However, our on-switching rate for Dronpa was smaller (0.029 s^−1^) compared to previously reported value (0.06 s^−1^). One plausible explanation to this discrepancy could be due to the difference in the environment of the PA-FP, since our values were measured in bacterial cells, and the reported value in mammalian cells. Interestingly, the on-rate for mNeonGreen was larger than for Dronpa (~2 fold), which explains the higher likelihood for spatially overlapping images of simultaneously activated localizations, manifested in enlarged localization widths (Fig. 2).

### Simple data post-processing to improve image quality reveals the organization of the bacterial cell division protein DivIVA and the chromosome organization protein ParB

As shown in the previous section, higher illumination power does not completely eliminate localizations arising from multiple mNeonGreen molecules emitting simultaneously (Fig. 2, Supplementary Fig. 2). Therefore, our PALM image of ParB-mNeonGreen contained localizations from single molecules as well as from multiple molecules, the latter likely masking the actual image which should comprise solely non-overlapping single molecules localizations. Next, we sought to implement a simple step of data post-processing to eliminate localizations from overlapping molecules. To this end, we have utilized the *B*. *subtilis* strain that expresses mNeonGreen fused to DivIVA. We did not use the ParB-mNeonGreen images because of the complexity needed to describe the *in situ* distribution of ParB, which is not ideal to evaluate the effect of data filtering. Contrary to ParB, the *in situ* location and organization of DivIVA has been described both with diffraction-limited and super-resolution microscopy^28^. DivIVA localizes at the division site and it assembles as two rings. These two rings can only be visualized with a super-resolution microscopy, such as structured illumination microscopy (SIM)^28^.

**Figure 5:**
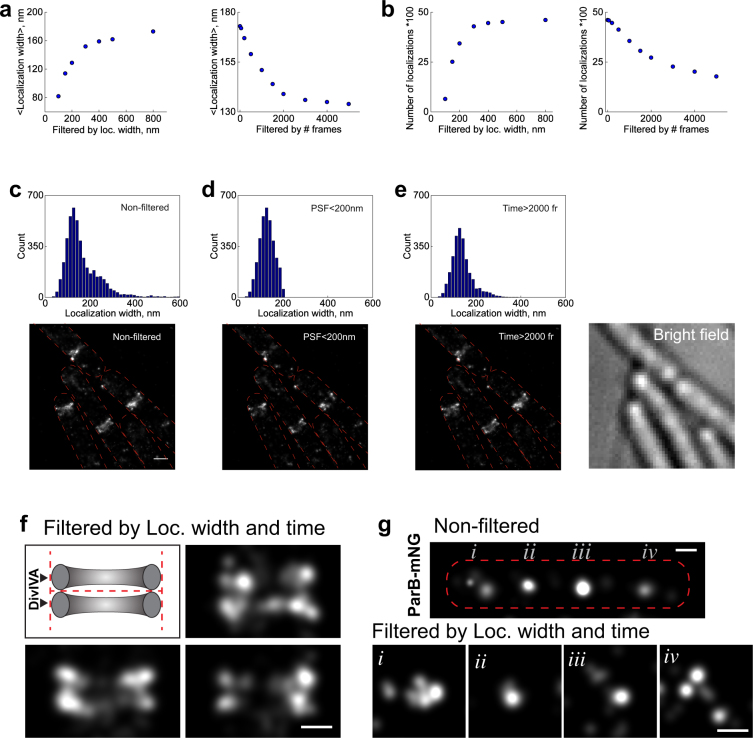
Effect of data filtering on the mNeonGreen PALM images **(a)** Mean localization width of detected mNeonGreen vs. filtering by localization width or number of frames from roi shown in **(c)** corresponding to *B*. *subtilis* expressing mNeonGreen fused to the DivIVA protein (BHF028). **(b)** Effect on the number of localizations when filtering either by localization width or by number of frames. **(c**,**d**,**e)** Histograms of localization widths before (c) and after filtering out localizations with widths larger than 200 nm (d) or frames corresponding to the first 2,000 frames (e). Localization width is the radius at which the fitted Gaussian drops to e^−1^ of its maximum. **(f)** Cartoon representation and zoomed-in examples of DivIVA-mNeonGreen double ring after data filtering. **(g)** PALM images of *B*. *subtilis* expressing ParB-mNeonGreen (BSG2204) before (top) and after (bottom) data filtering (both time and width). The filtered images are zoomed-in regions (*i*, *ii*, *iii and iv*) from the non-filtered image. Scale bar, 200 nm. Brightness of the PALM image are adjusted to emphasize description of the structure.

As expected, we also observed two bands (arising from the two-dimensional projections of two rings) when DivIVA-mNeonGreen was imaged with SIM (Supplementary Fig. 1a). Next, we imaged the DivIVA-mNeonGreen strain with the above described moderate illumination power. As expected, DivIVA-mNeonGreen showed double bands, however only in few cases. Instead, in some cases the “two bands” were blurry, and in many cases showed a structure that poorly resembled double bands (Fig. 5c, Supplementary Fig. 3b).

Next, we systematically evaluated the impact of data filtering on the DivIVA-mNeonGreen PALM images. Specifically, we evaluated the effect of removing localizations larger than a certain width or localizations appearing after a certain number of frames (Fig. 5a,b). With these filters, we expect to remove mostly localizations detected from overlapping emitters. First, we evaluated the effect of such data filtering in a limited region of interest (roi) in the DivIVA-mNeonGreen image (Fig. 5c), rather than on a whole field of view. As expected, the mean localization width decreases as the filter parameter becomes more stringent, *e*. *g*. smaller localization width or larger number of frames (Fig. 5a). Similarly, stringent filtering lowers the number of localizations (Fig. 5b). Therefore, it is important to choose the filter values wisely to avoid artefacts (see more in Discussion).

As mentioned earlier, optimization of the data acquisition step did not completely eliminate localizations arising from multiple overlapping molecules, which is corroborated also in our histogram of the localization width from mNeonGreen fused to DivIVA (Fig. 5c). Indeed, the population corresponding to the second peak at larger value contained around 30 percent of the total population (Supplementary Fig. 3a). This second population most likely represents localizations from multiple molecules, which ultimately may introduce artifacts to the PALM image. Data filtering by acquisition time (<2000fr), or width (>200 nm), mainly eliminated this second peak, and revealed the two bands (Fig. 5c,f, Supplementary Fig. 3b). Filtering data by localization width only showed limited improvements of revealing the two bands. As a control experiment we also created a *B*. *subtilis* strain that expresses DivIVA fused to the photoactivatable orange-emitting mCherry protein (pamCherry). In good agreement, our filtered DivIVA-mNeonGreen data and DivIVA-pamCherry showed similar double band structures (Supplementary Fig. 1b). It is important to note that our DivIVA images do not show two continuous bands as visualized with maximum intensity projection images from SIM (Supplementary Fig. 1a top). Because our PALM images are two-dimensional, focused at mid-cell and comparably smaller depth of field, they are rather similar to the SIM images at the mid-cell cross section (Supplementary Fig. 1a bottom).

Lastly, we show that filtering data of mNeonGreen fused to ParB indeed leads to multiple sub-clusters (Fig. 5g, Supplementary Fig. 4) similar to the ones observed with mEos3.2 fused to ParB (Fig. 1b). The biological implication of such organization is under study.

### Optimization of bacterial cell sample preparation

**Figure 6:**
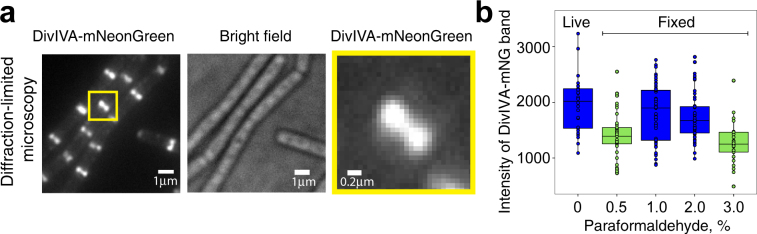
Effect of various paraformaldehyde concentrations on the fluorescence intensity and integrity of the DivIVA-mNeonGreen band. (**a**) Visualization of DivIVA-mNeonGreen using diffraction-limited microscopy. Microscopy samples of *B*. *subtilis* cells expressing DivIVA-mNeonGreen (BHF028) were prepared as described in Materials and Methods. (a, left) Fluorescence and (a, middle) bright field images of DivIVA-mNeonGreen expressing cells. (a, right) Zoomed-in region from “a, left” yellow. (**b**) Fluorescence intensity of the DivIVA-mNeonGreen bands, determined by drawing a line along the bands and extracting the peak value of the intensity profile along the line by using the software Fiji. Data are represented as a boxplot of at least 29 DivIVA-mNeonGreen bands from five different fields of view of one experiment for each condition. Significant differences between the samples were determined using a multiple comparison test (pairwise comparisons adjusted appropriately for multiple comparisons) after Kruskal-Wallis (P value: 0.05). Results are indicated in green or blue, in which conditions in same color represent no statistical difference. The horizontal line within each box indicate the median.

**Figure 7:**
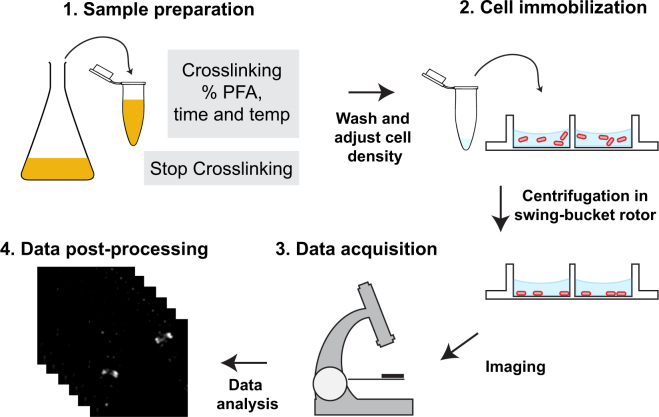
Workflow of bacterial sample preparation and PALM imaging. Workflow is divided into steps (1 to 4), each of which has been optimized in this work. Briefly, **(1)** upon harvesting cells and crosslinking, we add glycine to stop the crosslinking reaction. **(2)** After washing cells, the pellet is re-suspended in a volume which will yields an optimal cellular density. The cellular re-suspension is transferred onto a multi-well chamber (or spotted onto a coverslip) to be immobilized on the bottom of the glass. Cells are represented in red. **(3)** TIRF illumination and the illumination power should be adjusted by performing preliminary imaging. Finally, **(4)** post-data processing is required when utilizing mNeonGreen for PALM imaging.

Sample preparation is one of the crucial steps to generate artefact-free and high-quality microscopy images. Preserving the signal from fluorescence molecules is particularly essential for super-resolution microscopy because (a) the localization precision depends on the fluorescence intensity of the detected single molecules and (b) the accuracy of a molecular organization description depends on the labeling density. In this section, we describe some of the crucial steps for sample preparation (Figs 6, 7) of bacterial cells.

To select an optimal fixing condition, we have evaluated various parameters —such as the concentration of the crosslinker paraformaldehyde, buffer, temperature and duration of the crosslinking step— on the fluorescent intensity of DivIVA-mNeonGreen. Using diffraction-limited microscopy, we imaged live and fixed cells, and quantified the fluorescence intensity at the DivIVA-mNeonGreen band (Fig. 6). Fixation reduced the fluorescent intensity at various degrees, but because one percent paraformaldehyde fixation showed an intensity closest to the one from the live cell imaging (Fig. 6b), we chose 1% for our crosslinking protocol. We also evaluated the effect of crosslinking in buffer vs. in medium on the DivIVA-mNeonGreen as well as on ParB-mNeonGreen fluorescence intensity (Supplementary Fig. 6). We obtained the best results when cells are grown in a minimal medium and the crosslinker is directly added into the medium for DivIVA-mNeonGreen. In contrast, we obtained better results for ParB-mNeonGreen when cells were crosslinked in PBS (Fig. 6 and Supplementary Fig. 6). While the exact reason for this different observation is still to be explored, it seems to be important to optimize crosslinking conditions strain to strain.

## Discussion

In this study, we show how multiple simultaneously emitting mNeonGreen molecules hinder the use of mNeonGreen for super-resolution microscopy imaging. We identified an illumination power that minimizes the simultaneous emission of multiple nearby molecules and applied data post-processing to further decrease localizations raised from multiple emitters. Additionally, we described the impact of the various sample optimization steps to finally produce the most suitable sample for microscopy. We expect that our work can motivate scientists to further investigate the photo-chemical mechanisms of mNeonGreen switching, as well as to study the role of the chromosome organization proteins on the chromosome folding.

Our goal in this study was to establish a simple and practical method to utilize mNeonGreen fluorescent protein for PALM imaging. Because the reconstructed PALM image represents individually localized molecules, it is crucial to identify emissions rising from single molecules. Unlike other green PA-FPs utilized in PALM, the process of switching between the on (bright) and off (dark) states of mNeonGreen depends on a single wavelength (blue), which makes challenging to manipulate this switching process. Consequently, multiple molecules from a diffraction-limited area can be accumulated at the bright state emitting light simultaneously, which ultimately will be identified as a single molecule. Thus, the fitted localization center will be far from the actual individual molecule position. Because the probability of this situation occurring is higher at the beginning of the data acquisition (Fig. 2a), the developers of mNeonGreen illuminated the field of view with a strong laser power before the actual data acquisition initiated, which drives the molecules to the dark state^25^. However, such a strategy would also bleach molecules reducing the pool of mNeonGreen molecules, thus decreasing the number of localizations. Although this decrease might have a negligible impact when imaging high copy and densely labeled proteins, minimization of bleaching is important because of the big range of protein copy numbers in cells^39^. In addition, when imaging live cells one should balance photo-toxicity and the healthiness of the sample^40,41^. Our systematic evaluation of the effect of laser power on the simultaneous multiple emitters shows that the probability of multiple emission drastically decreases with increasing laser power, and it reaches a plateau at a medium power (Fig. 2b and Supplementary Fig. 2a). Therefore, we have chosen this medium power for our data acquisition. It is recommended to find a minimum laser power to drive the molecules to the dark state, thus minimizing bleaching that will decrease the pool of mNeonGreen molecules.

Properties of a PA-FP, such as photon budget, and on- and off- switching rate ratio will determine the image quality in superresolution microscopy^38,42^. *i)* The photon budget of mNeonGreen determined here (~669, Fig. 4) is in good agreement with the previously determined values (300–660^25^) and yielded a localization precision of ~27 nm. This precision was sufficient to distinguish the expected double band of DivIVA protein and also visualize subclusters of ParB for the first time with PALM (Fig. 5g and Supplementary Fig. 3). *ii)* The on-off switching ratio limits the density of fluorescent labels that can be localized properly^38,42^. This is particularly apparent in the case of DivIVA located at the division septum. Our off-rate for mNeonGreen was similar to the reported values of other green PA-FPs Dronpa and mGeosM. However, the on-rate was larger^38^, which supports the interpretation of localizations with larger width corresponding to multiple overlapping molecules. Thus, validating the necessity of implementing data post-processing to minimize the impact of artifacts due to overlapping single molecule images. An alternative technique to avoid high labelling density is to pre-bleach the sample with high laser power before acquiring data^1,25,43,44^. This pre-bleaching is essentially equivalent to a filtering step, similar to our post-processing step, to eliminate localizations in the beginning of the data acquisition, albeit without much control over what is filtered out.

Overlapping localizations due to multiple nearby mNeonGreen molecules simultaneously emitting light might deteriorate super-resolution image quality. One way to circumvent this issue is to use programs with multi-emitter fitting functions such as ThunderSTORM^45,46^ (SI Fig. 7). However, the method proposed here to apply data post-processing, to filter out those overlapping localizations by temporal information and spatial information (localization width) (Fig. 5), is extremely simple and quick, allowing one to troubleshoot experiments and data analysis with high turnover. Data filtering can be applied in the whole field of view or only to the region of interest. However, one should be careful when filtering data, since it can introduce artifacts (*e*.*g*. over-filtering) that may lead to misinterpretation of protein location and organization. To avoid misinterpretation, one should compare various parameters of data filtering. Complementary and if it is possible, the same target protein should be imaged with a different fluorescent protein to ensure integrity of protein location and organization.

Labeling of a target protein with a tag, in our case fluorescent proteins, could compromise the functionality of the target. Ideally, a tag should not interfere with the cellular localization nor the function of the tagged protein. In our study, we have created strains that expressed DivIVA fused to various PA-FPs, pamCherry, mNeonGreen, Dronpa and mGeosM. Unlike pamCherry and mNeonGreen fusion proteins (Fig. 5 and Supplementary Fig. 1), mGeosM and Dronpa mislocalized DivIVA preferentially at the poles (Fig. 3), possibly due to multimerization of the fusion proteins. Hence, it is advisable to evaluate the effect of fusion proteins for individual targets. Utilizing truly monomeric fluorophores like mScarlet^47^ or mNeonGreen^25^ can help avoid artifacts or non-desired phenotypes arising from artificial multimerization. Lastly, autofluorescence of the specimen can narrow down the options for PA-FPs even more. While orange emitting PA-FPs, *e*.*g*. pamCherry, mEos3.2, present properties for good quality superresolution imaging^27,38^, these PA-FPs would not be suitable for specimens with autofluorescence in orange/red (e.g. *Bacillus subtilis* and *Corynebacterium glutamicum*). In these cases, mNeonGreen could be an attractive option.

In conclusion, we have shown that we can enrich single mNeonGreen emitters by selecting a suitable illumination power, eliminating localizations arising from multiple simultaneous emitters, and optimizing sample preparation. We expect that our method allows the use of mNeonGreen for super-resolution microscopy and also contributes to the achievement of multi-color PALM images.

## Materials and Methods

### Sample preparation

#### Cell culture

*Bacillus subtilis* cells were grown overnight in NA medium (0.5% peptone, 0.2% yeast extract, 0.1% meat extract and 0.5% NaCl) at 30 °C to be diluted (1:100) the next morning into a minimal SMG medium (15 mM (NH_4_)_2_SO_4_, 61 mM K_2_HPO_4_, 44 mM KH_2_PO_4_, 3.4 mM sodium citrate 2xH_2_O, 1.7 mM MgSO_4_, 5.9 mM glutamate and 27 mM glucose) supplemented with 1.0 mM tryptophan. Cells were grown until OD_600_ reached ~0.15.

#### Cell fixation

A 0.9 mL portion of the cell culture was fixed gently rocking for 30 minutes at 37 °C in a solution containing 50 µL of 1 M sodium phosphate (pH 7.5) and paraformaldehyde (0.5, 1.0, 2.0 or 3.0% (w/v)). The reaction was stopped by adding glycine (15 mM final concentration) and further incubation for 10 min at 37 °C in a rocker. Then, the sample was centrifuged and the cellular pellet re-suspended in 50 µL of SMG medium (final OD_600_ is ~2). Cells expressing ParB-mNeonGreen or ParB-mEos3.2 were washed with PBS prior to fixation.

#### Cell immobilization

Fixed cells were immobilized either on poly-L-lysine coated coverslips or in multi-well chamber (μ-Slide Well Glass Bottom, Ibidi 80827). Coverslips or chamber were incubated with 1:10 diluted poly-L-lysine solution (Sigma P8920) for 30 min or overnight at 4 °C. Poly-L-lysine solution was removed and coverslips (or chamber) were rinsed three times with Milli-Q water. 50 µL of the fixed cells and 1 µL of *fiducial particles (40 nm diameter gold particles) were deposited on the slide (or into the chamber), and centrifuged for 10 min at 3,700 g. Non-immobilized cells were removed by washing three times with PBSG and cells were stored in 200 μl of PBSG until imaging. *Approximately 5 fiduciary particles were present per field of view.

### PALM imaging

PALM data was acquired and analyzed with a Zeiss Elyra P1 microscope and the accompanied Zen software. Samples expressing mNeonGreen fusion proteins were illuminated with a 488 nm laser, whose power varied from 46 to 763 W/cm^2^. Samples containing mEos3.2 or pamCherry fusion proteins were simultaneously illuminated with the excitation laser (561 nm, 202 W/cm^2^) and activation laser (405 nm). During data acquisition, the activation laser power was increased in multiple steps from 0.3 to 65 W/cm^2^). All samples were illuminated in pseudo-TIRF (total internal reflection fluorescence) mode and recorded at 20 Hz. All samples were imaged with the Zeiss objective alpha Plan-Apochromat 100×/1.46 Oil. Reported laser powers were measured at the sample plane. The mNeonGreen datasets were analyzed with the Zen software, with parameters “peak” and “peak mask size” set to 7 and 9, respectively. For the mEos3.2 data analysis, these values were 8 and 9, respectively. The localization precision reported here was determine using the Zen software, which calculates based on the equation in Thomson *et al*.^48^.

### Determination of on- and off-switching rates

Data acquisition and analysis to determine the on- and off- switching rates were performed as described earlier^38^. Briefly, bacillus cells expressing DivIVA-mNeonGreen or DivIVA-Dronpa were imaged in superresolution mode at a frame rate of 50 Hz. For DivIVA-Dronpa expressing cells, data acquisition started in the sole presence of the imaging laser (488 nm) (*i*.*e*. absence of the activation laser (405 nm)), followed by simultaneous illumination with imaging and activation laser until close to zero localization appeared per frame. In the case of DivIVA-mNeonGreen expressing cells, data was acquired only in the presence of the 488 nm laser (15% in the Zen software) since activation and imaging wavelengths are the same. At least 20,000 frames were acquired to ensure imaging of the PA-FPs to completion. On- and off-switching rates were obtained from localizations distributed in the cytoplasm (*i*.*e*. not from the highly concentrated regions such as cell poles and at the DivIVA bands).

## Electronic supplementary material

Supplementary Information 
