# Supplementary material for: Optimization of sample preparation and green color imaging using the mNeonGreen fluorescent protein in bacterial cells for photoactivated localization microscopy
Source: Sci Rep. 2018 Jul 4;8:10137. doi: 10.1038/s41598-018-28472-0 (PMC6031688; doi:10.1038/s41598-018-28472-0)
Supplement: Supplementary file 1 — Supplementary Information [file 41598_2018_28472_MOESM1_ESM.pdf]

Supplementary Information

Optimization of sample preparation and green color imaging using the mNeonGreen fluorescent protein in bacterial cells for photoactivated localization microscopy

Iris Stockmar[1,4,*], Helge Feddersen[2,*], Kimberly Cramer[1,4], Stephan Gruber[3], Kirsten Jung[1], Marc Bramkamp[2,#], Jae Yen Shin[1,4,#]

[1]Munich Center for Integrated Protein Science (CIPSM) at the Department of Biology I, Microbiology, Ludwig-Maximilians-Universität München, Martinsried, Germany

[2]Department of Biology I, Microbiology, Ludwig-Maximilians-Universität München, Martinsried, Germany

[3]Department of Fundamental Microbiology, University of Lausanne, Switzerland

[4]Present address: Max Plank Institute for Biochemistry, Germany

*Equally contributed

[#]Corresponding authors: jshin@biochem.mpg.de (J.Y.S.); marc.bramkamp@lmu.de (M.B.)

Supplementary Table

All strains were constructed for this study.

All strains are auxotrophic for tryptophan (*trpC2*).

**Strain**	**Genotype or description**	**Background strain**
BSG2204	*parB::parB-mNeonGreen (spec)*	*B. subtilis* 1A700 provided by the **BGSC** (Bacillus Genetic Stock Center)
BSG2205	*parB::parB-mEos3.2 (spec)*	
BHF028	*divIVA::divIVA-mNeonGreen (spec)*	*B. subtilis* BGSC 1A1
JB37	*divIVA::divIVA-pamCherry (spec)*	
BHF057	*divIVA::divIVA-dronpa*	
BHF058	*divIVA::divIVA-mGeosM*	

SIM imaging

Three-dimensional structured illumination microscopy was performed with a DeltaVision OMX V3 microscope (GE), equipped with a 100 x/1.40 NA PlanApo oil immersion objective (Olympus), Cascade II:512 EMCCD cameras (Photometrics) and lasers for 405nm, 488nm and 594nm. Image stacks were first reconstructed and corrected for color shifts with the softWoRx 6.0 Beta 19 (unreleased) software. After establishing composite tiff stacks with a custom-made macro in Fiji, data were subsequently aligned again and maximum intensity projections were used.

Cell immobilization

The ability to generate monolayer of cells on the coverslip or chamber surface is another crucial factor when imaging bacterial cells and it mainly depends on the density of cells and the efficiency of cell immobilization on the surface. Although low cell density will guarantee a monolayer, it is impractical due to a huge amount of time invested in finding cells on the microscope as well as the low throughput due to low number of cells

per field of view. Therefore, one should aim to generate a field of view where cells are separated enough to be distinguished as individual cells and yet populated enough to image a high number of cells (Supplementary Fig. 6b). Once the cell density is adjusted, we spot (or add) the cells onto the coverslip (or chamber), incubate shortly and centrifuge using a swing-bucket rotor (Fig. 7). Usually we use the multi-well chamber to optimize sample preparation conditions, and then prepare our samples on the individual coverslips for the final super-resolution microscopy experiments.

SI Figure 1

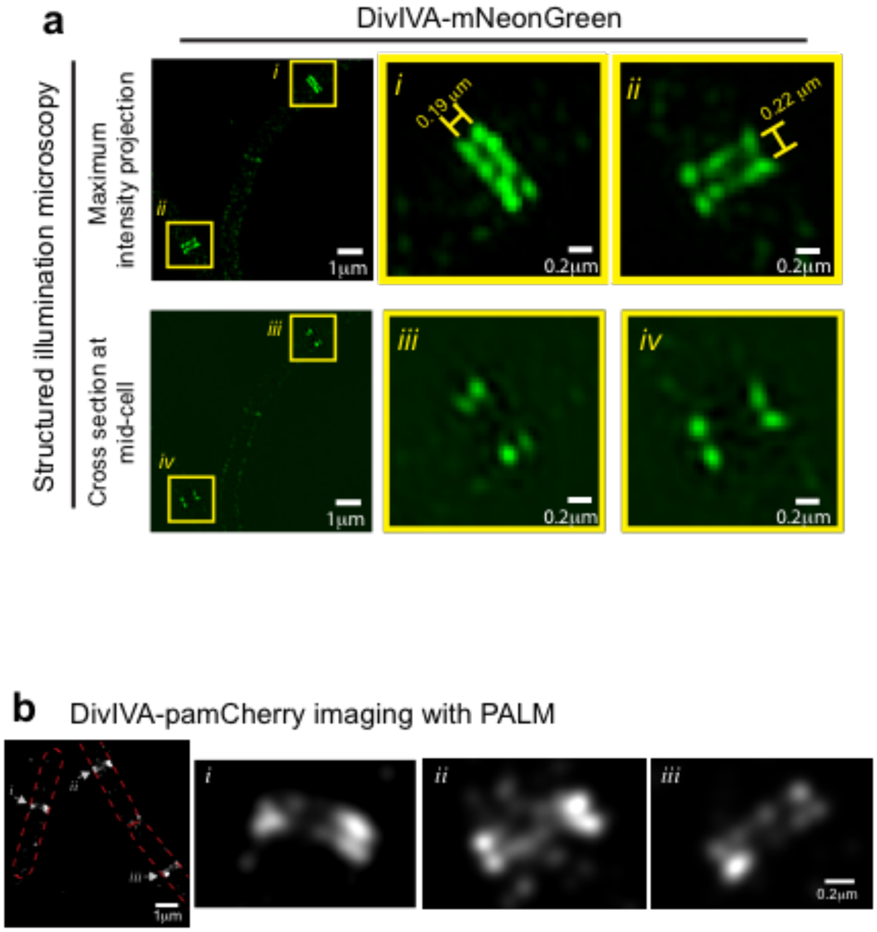

SI Figure 1. Super-resolution images of DivIVA from *B. subtilis*. (a) Structured Illumination Microscopy (SIM) shows that DivIVA-mNeonGreen organizes as double bands. The distances (0.19 and 0.22μm) between the double bands were similar to the values reported previously by Eswarammorthy *et.al*. 2011. Maximum intensity projection and mid-cell cross section are shown from the same region of interest. **(b)** PALM microscopy of DivIVA. Microscopy samples of *B. subtilis* cells expressing DivIVA-pamCherry (JB37) were prepared, PALM images were taken and analyzed as described in Materials and Methods. Regions of interest *"i"* to *"iii"* from the left images were cropped, zoomed-in, and brightness and contrast adjusted using the program Fiji.

SI Figure 2

Histogram of localization width at various 488nm power

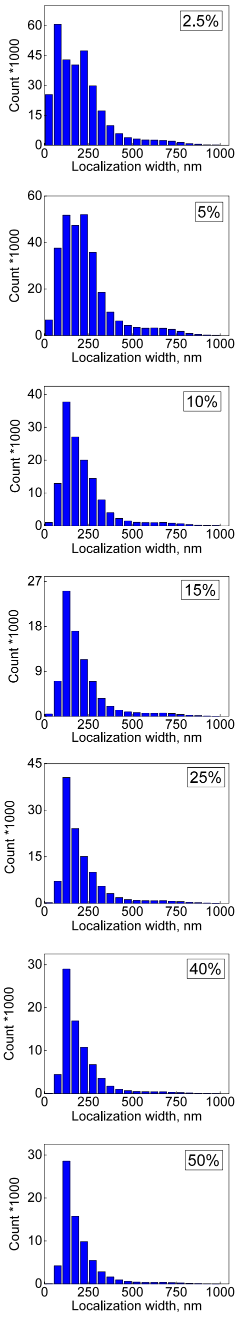

SI Figure 2. Histogram of mNeonGreen localization width from DivIVA-mNeonGreen expressing cells. Localization width of all detected mNeonGreen from the whole field of view was utilized to plot the histograms.

PSF FWHM according to Raleigh is 0.61 λ/na (λ is the wavelength and na is the numerical aperture of the objective). The here reported localization width is the distance

of PSF center to the radius at which the intensity drops to 1/e. Hence, the theoretical expectation of the reported width is $\frac{\sqrt{2}}{2.355} * \frac{0.61\lambda}{\text{na}}$. With λ of 514nm (for mNeonGreen), the expected value is 129nm. This value matches quite well the position of the histogram maxima (125nm). We suspect that the skewness of the histogram towards larger number is a result of overlapping localizations. Due to the skewness, mean values in Fig 2b are larger than the theoretically expected localization width. In fact, a double Gaussian fit of SI Fig. 3 results in a peak center at 123nm for the population corresponding to the smaller localization width, in a good agreement to the theoretical value.

SI Figure 3

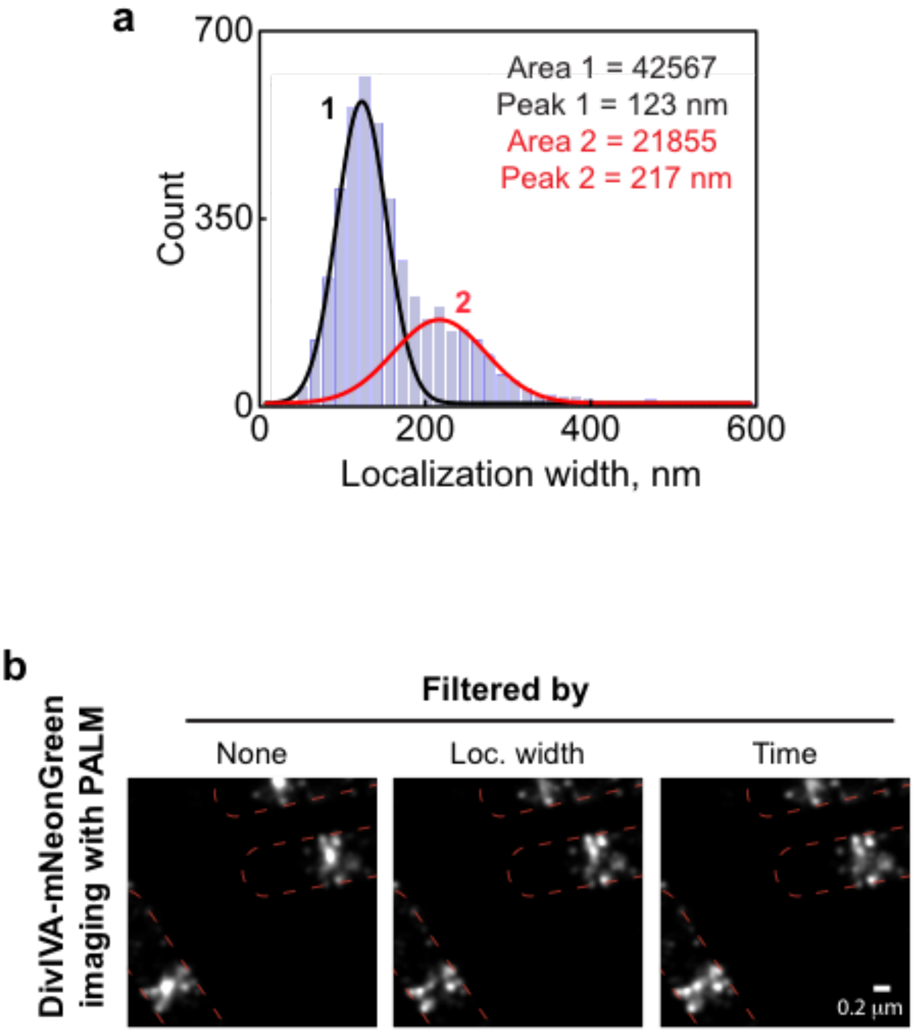

SI Figure 3. (a) Histogram of mNeonGreen localizations width of DivIVA-mNeonGreen expressing bacillus cells. Distribution was fitted to two Gaussians. Area and peak values are indicated for both populations. **(b) Effect of data filtering on the DivIVA-mNeonGreen PALM images.** Microscopy samples of cells expressing DivIVA-mNeonGreen (BHF028) were prepared, and PALM images were taken and analyzed as described in Materials and Methods. Brightness of the PALM image are adjusted to emphasize description of the structure.

SI Figure 4

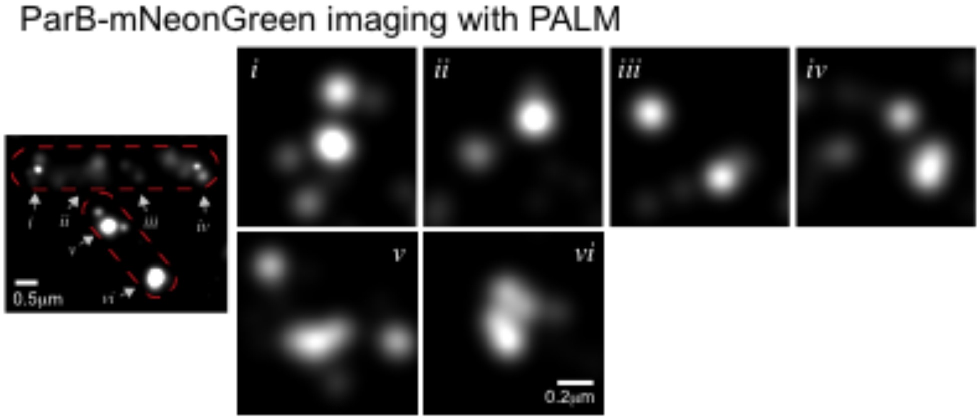

SI Figure 4. Visualization of ParB proteins using PALM. Microscopy samples of *B. subtilis* cells expressing ParB-mNeonGreen (BSG2204) were prepared, and PALM images were taken as described in Material and Methods. Analyzed data were filtered using the Elyra microscope program Zen. Details of filtering parameters are described in Material and Methods. Regions of interest *"i"* to *"vi"* from the left images were cropped, zoomed-in, and brightness and contrast adjusted using the program Fiji.

SI Table I

Table 1. Properties of Dronpa and mNeonGreen PAFPs

**PAFP**	**On-switching rate, s^{-1}**	**Mean life time, s**	**On-off switching rate ratio**
Dronpa	0.029 ($5e^{-5}$)	0.009 ($6e^{-4}$)	2.5×10^{-4}
mNeonGreen	0.056 ($1.3e^{-4}$)	0.012 ($3.4e^{-4}$)	6.9×10^{-4}

These data were obtained from *B. subtilis* expressing DivIVA fused to either Dronpa or to mNeonGreen fluorescent protein.
Reported errors in parenthesis are standard errors of the fitted parameters
For details see Material and Methods

SI Figure 5

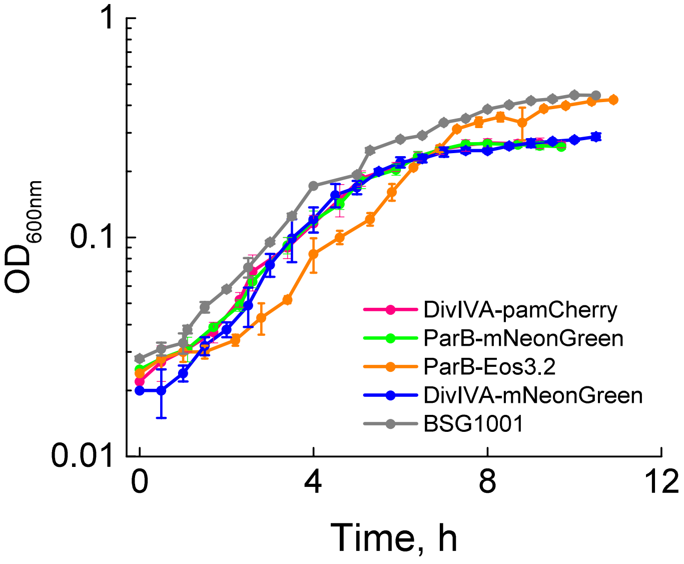

SI Figure 5. Growth curves for *Bacillus subtilis* cells expressing the indicated fusion proteins. Cell either expressing DivIVA-pamCherry (JB37), DivIVA-mNeonGreen (BHF028), ParB-mNeonGreen (BSG2204), ParB-mEos3.2 (BSG2205) and wildtype.

(BSG1001) were grown at 30°C in SMG minimal medium. Optical density at 600nm was monitored every 0.5 h. Error bars represent the standard deviation of three independent experiments.

SI Figure 6

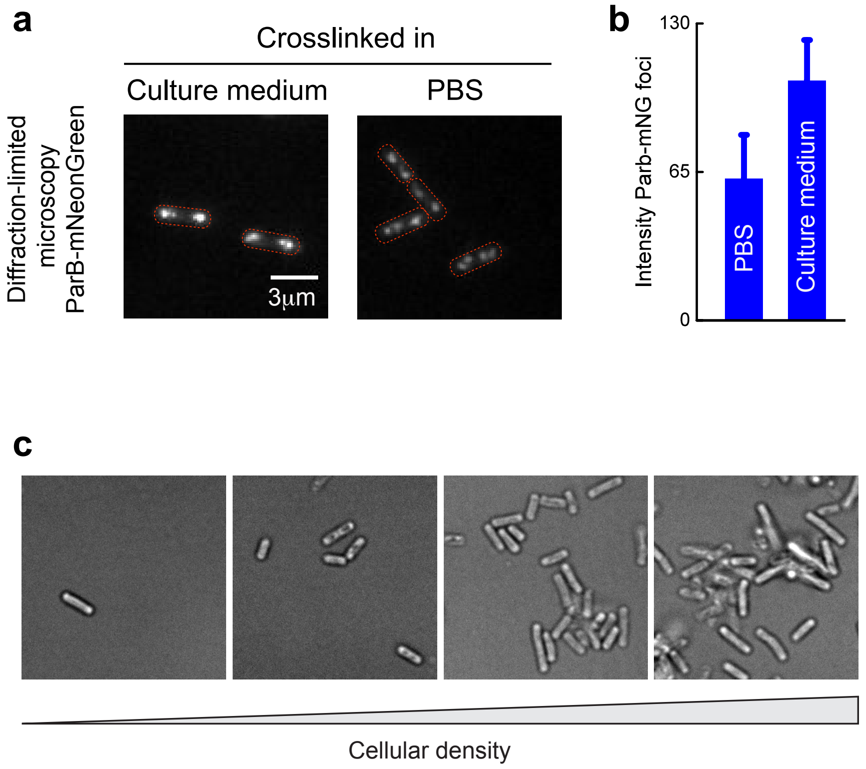

SI Figure 6. (a) Effect of crosslinking on the fluorescence intensity of Parb-mNeonGreen foci intensity. Representative diffraction limited microscopy images of *B. subtilis* cells expressing Parb-mNeonGreen (BSG2204) crosslinked directly in the medium and in PBS buffer. **(b)** Fluorescence intensity of the ParB-mNeonGreen foci was determined by drawing a line along the cell and extracting the peak value of the intensity profile along the line by using the software Fiji. Bar graph represents the mean fluorescence intensity of at least 40 foci and their standard deviation. **(c) Effect of cellular density on the microscopy slide preparation.** Bright field images of microscopy samples of *B. subtilis* cells expressing ParB-mNeonGreen (BSG2204). Cells were harvested, fixed and adjusted to various (0.5, 1.5, 3.0 and 5.0) OD_{600nm}. 50μL of these cells were prepared as described in Materials and Methods. As the cellular density increases, higher number of cells are attached on the glass surface to the extent that multilayer of cells is generated. Monolayer is ideal for cellular imaging, since multilayer generates non-desired background and it is hard to properly assign signals to the corresponding cell.

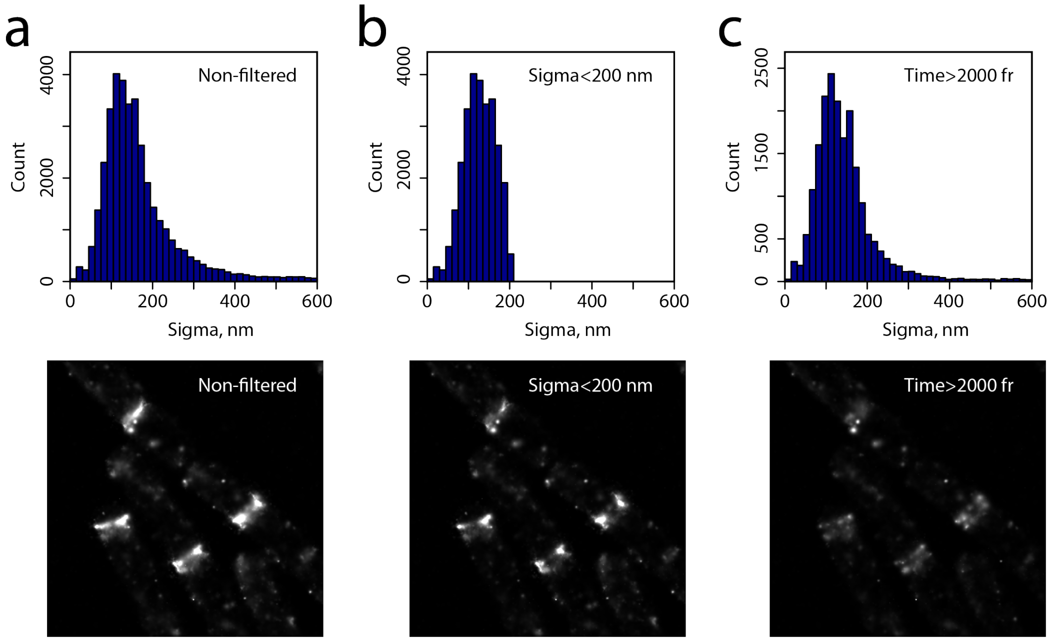

SI Figure 7. mNeonGreen PALM data analyzed using the multi-emitter fitting function of ThunderSTORM. Data from the same region of interest from Fig. 5 was analyzed with ThunderSTORM[1], using the multi-Gaussian PSF fitting method (maximum likelihood with enabled multi-emitter fitting). Histograms of the reported localization width (sigma) **(a)** before and after filtering out localizations with **(b)** sigma larger than 200nm or **(c)** frames corresponding to the first 2,000 frames**.** Corresponding PALM images are shown. Sigma is the standard deviation of the probability density of the fitted 2D Gaussian function.

Reference:

1. Ovesný, M., Křížek, P., Borkovec, J., Svindrych, Z. & Hagen, G. M. ThunderSTORM: a comprehensive ImageJ plug-in for PALM and STORM data analysis and super-resolution imaging. *Bioinformatics* **30,** 2389–2390 (2014).
